# NGSReadsTreatment – A Cuckoo Filter-based Tool for Removing Duplicate Reads in NGS Data

**DOI:** 10.1038/s41598-019-48242-w

**Published:** 2019-08-12

**Authors:** Antonio Sérgio Cruz Gaia, Pablo Henrique Caracciolo Gomes de Sá, Mônica Silva de Oliveira, Adonney Allan de Oliveira Veras

**Affiliations:** 1 Postgraduate Program in Applied Computing, Federal University of Pará (UFPA), Pará, Brazil; 2 Federal Rural University of Amazonia Campus Tomé-Açu (UFRA), Pará, Brazil

**Keywords:** Computational platforms and environments, Software

## Abstract

The Next-Generation Sequencing (NGS) platforms provide a major approach to obtaining millions of short reads from samples. NGS has been used in a wide range of analyses, such as for determining genome sequences, analyzing evolutionary processes, identifying gene expression and resolving metagenomic analyses. Usually, the quality of NGS data impacts the final study conclusions. Moreover, quality assessment is generally considered the first step in data analyses to ensure the use of only reliable reads for further studies. In NGS platforms, the presence of duplicated reads (redundancy) that are usually introduced during library sequencing is a major issue. These might have a serious impact on research application, as redundancies in reads can lead to difficulties in subsequent analysis (e.g., *de novo* genome assembly). Herein, we present NGSReadsTreatment, a computational tool for the removal of duplicated reads in paired-end or single-end datasets. NGSReadsTreatment can handle reads from any platform with the same or different sequence lengths. Using the probabilistic structure Cuckoo Filter, the redundant reads are identified and removed by comparing the reads with themselves. Thus, no prerequisite is required beyond the set of reads. NGSReadsTreatment was compared with other redundancy removal tools in analyzing different sets of reads. The results demonstrated that NGSReadsTreatment was better than the other tools in both the amount of redundancies removed and the use of computational memory for all analyses performed. Available in https://sourceforge.net/projects/ngsreadstreatment/.

## Introduction

The advent of Next-Generation Sequencing (NGS) technologies in mid-2005 provided significant breakthroughs in the omics fields. These platforms can generate millions of reads in a short time; for instance, Illumina NextSeq is capable of generating 400 million reads per round. The genomic library must be prepared prior to the actual sequencing and one task included in this stage is polymerase chain reaction (PCR) amplification^1^.

PCR generates a super-representation of a sample fragment, giving rise to the concept of coverage, where the genetic material of an organism to be sequenced presents a several-fold multiplication of its expected size. This super-representation is important for several analyses, including frameshift curation and single nucleotide polymorphism analyses, among others^1^.

However, some analyses are impacted by this super-representation, such as *de novo* assembly and the final scaffolding process. Moreover, the tasks demand a high computational cost, and duplication gives rise to false positives with overlapping contigs, as well as their subsequent extension due to the high number of connections. Consequently, false negatives arise as a result of the overlapping conflicts generated by the duplications^2^.Thus, the development of computational methods that can remove sequencing read redundancies is important. Several software solutions have been developed over the years to address this situation. GPU-DupRemoval (by Removing GPU Duplicates) aims to remove duplicate reads using graphical processing units (GPUs) generated with the Illumina platform. The task is divided into two phases: the clustering of possible duplicate sequences according to their prefix, followed by comparison of the sequence suffixes in each cluster to detect and remove redundancies^3^.

The FASTX-Toolkit Collapser (http://hannonlab.cshl.edu/fastx_toolkit), FastUniq^4^, Fulcrum^5^, and CD-HIT^6^ tools employ an alignment-free strategy. FASTX-Toolkit Collapser is able to identify and remove identical sequences from single-end reads. FastUniq, on the other hand, is designed to remove identical duplicates in three steps: initially, all paired reads are loaded into the memory; subsequently, the read pairs are sorted, and finally the duplicate sequences are identified by comparing the adjacent read pairs in the sorted list.

Fulcrum is able to identify duplicates that are fully or partially identical. Reads identified as possible duplicates are kept in different files, whose maximum size is defined by the user. The read sequences within each file are compared to identify duplicates^5^.

CD-HIT has two different tools for removing duplicates of single-end and paired-end reads generated with the Illumina platform. CD-HIT-454 parses libraries generated with 454 to identify exactly identical duplicates^6^.

The majority of the existing tools are designed to serve a particular sequencing platform. Thus, we present the NGSReadsTreatment tool for the removal of read redundancies for any NGS platform, based on the probabilistic structure of Cuckoo Filter.

## Results and Discussion

The reads sets of the sixteen organisms (real datasets) were processed using the FastUniq 1.1^4^, ParDRe 2.2.5^7^, MarDre 1.3^8^, CD-HIT-DUP 4.6.86^6^, Clumpify (https://sourceforge.net/projects/bbmap), and NGSReadsTreatment computational tools. The percentage of redundancy removal for each organism as well as an evaluation of the total memory used per tool is shown in Tables 1 and 2, respectively.

**Table 1:** Percentage of read redundancy removal per tool for each organism. NP - not processed owing to errors.

Organism	FastUniq 1.1	ParDRe 2.2.5	MarDre 1.3	CD-HIT-DUP 4.6.8	Clumpify (bbmap)	NGSReadsTreatment
SRR2014554	NP	NP	NP	NP	NP	0.29%
ERR007646	50.55%	50.55%	50.55%	50.55%	50.65%	50.50%
SRR2000272	0.82%	0.81%	NP	0.81%	0.95%	1.91%
SRR1424625	0%	0%	0%	0%	0.20%	0.94%
SRR933487	0.72%	0.72%	0.72%	0.72%	1.11%	1.90%
SRR6479489	0.14%	0.14%	0.13%	0.14%	0.19%	1.21%
SRR6479482	0.14%	0.14%	0.14%	0.14%	0.18%	1.17%
SRR974839	48.74%	48.74%	48.74%	48.74%	49.07%	49.08%
SRR1144800	0.06%	0.06%	0.06%	0.06%	0.10%	0.94%
SRR7587111	NP	0.37%	0.37%	0.37%	0.43%	0.87%
SRR7819959	NP	0.74%	0.74%	NP	0.80%	1.36%
ERR2375157	NP	0.07%	0.07%	NP	0.08%	1.55%
SRR6799098	NP	2.11%	2.11%	2.1%	2.22%	2.22%
SRR7905974	NP	0%	NP	NP	0%	0.13%
SRR7739756	NP	0%	NP	NP	0%	0.08%
ERR2162181	NP	0%	0%	0%	NP	0.33%

**Table 2:** Memory amount used by each tool in megabyte. NP - not processed owing to errors.

Organism	FastUniq 1.1	ParDRe 2.2.5	MarDre 1.3	CD-HIT-DUP 4.6.8	Clumpify (bbmap)	NGSReadsTreatment
SRR2014554	NP	NP	NP	NP	NP	549
ERR007646	3987	5387	1653	5393	870	543
SRR2000272	1722	2278	NP	3076	423	537
SRR1424625	2571	3586	1676	4097	455	539
SRR933487	1449	1950	1411	2063	2215	538
SRR6479489	2629	3629	1652	4313	3454	538
SRR6479482	2783	3850	1647	4501	3616	538
SRR974839	2725	3825	1634	4499	2625	540
SRR1144800	2633	3730	1653	4351	3250	540
SRR7587111	NP	888	1118	1561	769	533
SRR7819959	NP	3197	1665	NP	659	537
ERR2375157	NP	1989	1394	NP	744	537
SRR6799098	NP	247	913	481	373	531
SRR7905974	NP	3796	NP	NP	961	526
SRR7739756	NP	1704	NP	NP	700	532
ERR2162181	NP	625	947	779	NP	535

Table 1 shows that NGSReadsTreatment obtained a greater percentage of redundant read removals for thirteen of the sixteen organisms analyzed, being that in an organism the percentage of removal equal to that of another tool used in the test; that is, it was able to identify and remove the largest amount of redundancies. Some datasets of organisms, for example SRR2000272, SRR7905974 and SRR2014554, experienced processing problems with the other computational tools: computer crashes during execution and processing failure due to the existence of orphan sequences in the read files. The tools that presented 0% were not able to remove any redundancy in the dataset, despite processing the data normally.

For the SRR2014554 organism, only the NGSReadsTreatment was successful in processing the 4-GB dataset. All the other tested tools presented errors during read processing.

Table 2 lists the total memory used by each tool in the processing of the raw reads. Similar to the results described in Table 1, the dataset of some organisms presented problems during the execution by the other tools. However, it was possible to use the NGSReadsTreatment software in all cases, thereby also demonstrating its efficiency in the use of memory, since it was the only tool that used the least computational memory among all the tested tools in most analyses.

The FastUniq software does not support single-end reads in its analyzes, so it was not possible to perform the processing of reads of this type with the tool. However, in all cases it was possible to use the NGSReadsTreatment, also demonstrating its efficiency in processing paired-end and single-end reads, with a reduced computational memory usage.

To improve the validation of NGSReadsTreatment the same analyzes performed with the real datasets (sixteen organisms) were performed with simulated datasets from ART tool^9^. It can be observed that NGSReadsTreatment has proved to be efficient for both redundancy removal and memory usage as shown in the Tables 3 and 4.

**Table 3:** Percentage of read redundancy removal per tool for each simulated dataset. NP - not processed owing to errors.

Organism	FastUniq 1.1	ParDRe 2.2.5	MarDre 1.3	CD-HIT-DUP 4.6.8	Clumpify (bbmap)	NGSReadsTreatment
*Mycobacterium tuberculosis variant bovis BCG* str. Korea 1168P - Platform HiSeq 2500	0.08%	NP	0.08%	0.08%	0.20%	0.77%
*Mycobacterium tuberculosis KZN* 4207 - Platform HiSeq 2500	0.08%	NP	0.08%	0.08%	0.20%	0.05%
*Escherichia coli* O103:H2 str. 12009 - Platform HiSeq 2500	0.09%	NP	0.09%	0.09%	0.23%	1.15%
*Arcobacter halophilus* strain CCUG 53805 - Platform HiSeq 2500	0.08%	NP	0.08%	0.08%	0.22%	0.10%
*Mycobacterium tuberculosis variant bovis BCG* str. Korea 1168P - Platform 454	0%	NP	NP	0%	0%	1.16%
*Mycobacterium tuberculosis KZN* 4207 - Platform 454	0%	NP	NP	0%	0%	0.08%
*Escherichia coli* O103:H2 str. 12009 - Platform 454	0%	NP	NP	0%	0%	1.43%
*Arcobacter halophilus* strain CCUG 53805 - Platform 454	0%	NP	NP	0%	0%	0.13%

**Table 4:** Memory amount used by each tool in megabyte for each simulated dataset. NP - not processed owing to errors.

Organism	FastUniq 1.1	ParDRe 2.2.5	MarDre 1.3	CD-HIT-DUP 4.6.8	Clumpify (bbmap)	NGSReadsTreatment
*Mycobacterium tuberculosis variant bovis BCG* str. Korea 1168P - Platform HiSeq2500	1272	NP	1474	2173	771	537
*Mycobacterium tuberculosis KZN* 4207 - Platform HiSeq2500	1278	NP	1533	2153	538	538
*Escherichia coli* O103:H2 str. 12009 - Platform HiSeq2500	1583	NP	1632	2660	558	537
*Arcobacter halophilus* strain CCUG 53805 - Platform HiSeq2500	832	NP	1250	1363	569	536
*Mycobacterium tuberculosis variant bovis BCG* str. Korea 1168P - Platform 454	143	NP	NP	477	337	534
*Mycobacterium tuberculosis KZN* 4207 - Library 454	143	NP	NP	476	262	534
*Escherichia coli* O103:H2 str. 12009 - Platform 454	177	NP	NP	598	400	533
*Arcobacter halophilus* strain CCUG 53805 - Platform 454	96	NP	NP	321	264	536

Most errors were observed during the processing of the single-end reads, all details on the errors and all processing results per organism are available in the supplementary material.

In the third validation step, after the generation of the nine datasets with different coverage, the reads were counted to determine the amount of reads, number of unique reads and the amount of redundant reads in the raw data of each dataset (last table of the section simulated data with different coverage values in the Supplementary Material).

All nine datasets were processed by all tools for redundancy removal, where the memory usage by each tool was evaluated. After this processing, the unique reads of each of the datasets were counted. This count seeks to identify whether the number of unique reads in a processed dataset (Supplementary Material) is equal to the number of unique reads of the raw dataset, thus ensuring that only redundant reads were removed in the analysis.

As can be seen in Supplementary Material, the NGSReadsTreatment and all the tools used, with the exception of the Clumpify (bbmap) tool, were able to reach the number of unique reads equal to the raw data, thus ensuring that all these tools succeeded in removing only the redundant reads of each dataset.

The Clumpify (bbmap) tool was the only one that presented a different number of unique reads in relation to the raw data, indicating that this tool may be removing more data than just redundant reads.

**Figure 1:**
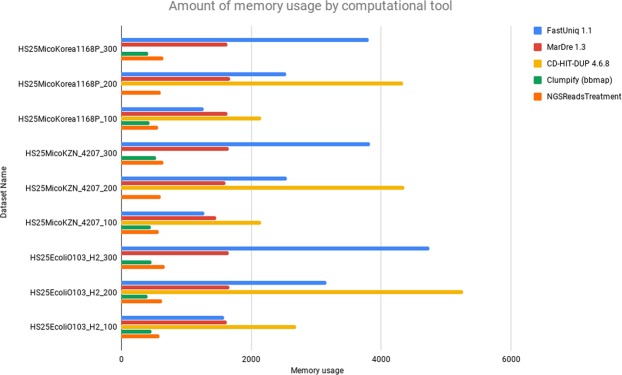
Evaluation of memory usage for each computational tool in the processing of simulated datasets.

As there was no difference in the amount of redundant reads removed between NGSReadsTreatment and the other tools of this analysis, with exception of the Clumpify (bbmap) tool, we can validate that all are removing only redundant data from the datasets, however, it is possible to observe the disparity in the amount of memory required for the data processing between NGSReadsTreatment and the other tools, where NGSReadsTreatment used a much smaller amount of memory to process the same amount of data (Fig. 1). All processing results per organism are available in the supplementary material.

The analysis of the results obtained herein allowed verification of the efficiency of the adopted Cuckoo Filter probabilistic data structure, as it proved effective in removing read redundancies from the raw files, besides showing optimal memory usage for task processing. The NGSReadsTreatment tool is capable of handling single-end and paired-end files, and is available in two versions: one with a graphical interface and control of processing status through a database. Thus, in case of some kind of error or if the user wishes to interrupt processing, it can be resumed. A version without a graphical interface is also available.

The NGSReadsTreatment presented the same behavior in the analysis of both real data and simulated data. The simulated dataset results show the efficiency of the NGSReadsTreatment in the removal of the reads redundancies as listed in Table 3.

Thus, it is concluded that NGSReadsTreatment has proven to be an efficient tool in removing redundancy from NGS reads, thus being an alternative in the execution of this task even if the user does not have high computational resources.

## Methodology

### Programming language and database

NGSReadsTreatment was developed in JAVA language (http://www.oracle.com/) and the Swing library was used to create the graphical interface (http://www.oracle.com/). Maven (https://maven.apache.org/) was used for dependency management and build automation. Its main features include the following (among others): simplified project configuration following best practices, automated dependency management, and JAR generation with all the dependencies used in the project. The project management was performed with SQLite version 3 (https://www.sqlite.org/).

### Redundancy removal

Cuckoo Filter^10^ was used to remove redundancies from the reads in the raw files. It is a quick and effective probabilistic data structure for cluster association queries. Developed by Fan, Andersen, Kaminsky, and Mitzenmacher, Cuckoo Filter emerged as an enhancement to Bloom Filter^11^, introducing support for dynamic item deletion, improved search performance, and improved space efficiency for low false-positive applications.

The Cuckoo Filter uses cuckoo hashing^12^ to resolve collisions and basically consists of a compact cuckoo hash table that stores the fingerprints of inserted items. Each fingerprint is a string of bits derived from the hash of the item to be inserted.

A cuckoo hash table consists of a two-dimensional array where the rows correspond to the associative units called buckets and their cells are called slots. A bucket can contain multiple slots and each slot is used to store a single fingerprint of predefined size^10^. For example a cuckoo filter (2,4) has slots that store 2-bit fingerprints and each table bucket can hold up to 4 fingerprints.

In the process of removing redundancy is generated for each read a fingerprint and checked if it is contained in the cuckoo hash table, if the answer is false the fingerprint is inserted into the table and the read is stored in a text file, otherwise the read is discarded.

It is worth mentioning that these probabilistic structures^10^ do not provide false negatives, which allows greater efficiency in the removal of duplicate reads from the raw file.

### Evaluation of computational cost

Linux’s *time* software (http://man7.org/linux/man-pages/man1/time.1.html) was used to generate statistics for a command, shell script, or any executed program. The statistics included the time spent by the program in the user mode, the time spent by the program in the kernel mode, and the average memory usage by the program. The output was formatted using the -f option or the TIME environment variable. The string type format was interpreted in the same way as *printf*, where common characters were copied directly whereas special characters were copied using \t (tab) and \n (new line). The percent sign is represented by %% (otherwise, % indicates a conversion^13^).

### Raw data download

Fastq-dump version 2.9.2 (https://edwards.sdsu.edu/research/fastq-dump/) was used to download the NCBI-SRA database files in fastq format.

### Tool validation with real datasets

To validate NGSReadsTreatment were used data from sixteen organisms. Two strains of *Mycobacterium tuberculosis*, two *Kineococcus*, six strains of *Escherichia* coli, and one strain of *Rhodopirellula báltica*, *Arcobacter halophilus*, *Rathayibacter tritici*, *Salmonella entérica*, *Staphylococcus aureus* and *Pseudomonas aeruginosa*. Each organism with its SRA number is listed in Table 5. For paired reads the File size by Dataset and Total of Reads by Dataset represent the sum of tag1 and tag2 (Table 5).

**Table 5:** Organisms and SRA number used to validate NGSReadsTreatment.

Organism	SRA Access number	File size by Dataset	Total of Reads by Dataset	Type Library	Platform
*Escherichia coli* RR1	SRR2014554	8192MB	24248885	Paired	Illumina HiSeq 2000
*Escherichia coli* 042	ERR007646	2406MB	14110696	Paired	(Illumina Genome Analyzer
*Escherichia coli* P12b	SRR2000272	1350MB	2990758	Paired	Illumina MiSeq
*Escherichia coli* KLY	SRR1424625	1682MB	6886668	Paired	Illumina HiSeq 2000
*Escherichia coli* O25b:H4-ST131	SRR933487	1070MB	3214312	Paired	Illumina Genome Analyzer IIx
*Kineococcus rhizosphaerae* DSM 19711	SRR6479489	2048MB	5641334	Paired	Illumina HiSeq 2500
*Kineococcus xinjiangensis* DSM 22857	SRR6479482	2168MB	5971022	Paired	Illumina HiSeq 2500
*Mycobacterium tuberculosis* F11	SRR974839	1936 MB	7279254	Paired	Illumina HiSeq 2000
*Mycobacterium tuberculosis XDR KZN 4207*	SRR1144800	1884MB	7033428	Paired	(Illumina HiSeq 2000
*Arcobacter halophilus*	SRR7587111	588MB	670813	Paired	454 Titanium
*Rhodopirellula baltica*	SRR7819959	1984MB	3207713	Single	Ion Torrent
*Escherichia coli O157:H7 in Romania*	ERR2375157	1201MB	2106268	Single	Ion Torrent
*Rathayibacter tritici*	SRR6799098	157MB	160403	Single	454 Junior
*Salmonella enterica*	SRR7905974	2990MB	163468	Single	PacBio
*Staphylococcus aureus*	SRR7739756	1336MB	86389	Single	Oxford nanopore MinIon
*Pseudomonas aeruginosa*	ERR2162181	246MB	1146696	Single	SoliD 5500

### Tool validation with simulated datasets

Aiming to further validate the tool NGSReadsTreatment another approach was employed, the use of simulated NGS datasets. The idea is that the tool NGSReadsTreatment should exhibit the same behavior in both real and simulated data.

To generate the simulated datasets, the ART tool version 2.5.8^9^ was used, which is able to generate simulated next-generation reads from different platforms, based on a reference in the fasta format. The ART tool can simulate real sequencing read errors and quality, and it is used to test or benchmark a variety of method or tools for next-generation sequencing data analysis.

For this validation of the NGSReadsTreatment were simulated reads from sequencing on the Illumina HiSeq 2500 and Roche 454 GS FLX Titanium platforms.

The organisms used as reference to generate the simulated reads were: *Mycobacterium bovis* BCG str. Korea 1168P (GenBank: CP003900.2), *Mycobacterium tuberculosis* KZN 4207 (GenBank: CP001662.1), *Arcobacter halophilus* strain CCUG 53805 (GenBank: CP031218) and *Escherichia coli* O103:H2 str. 12009 (GenBank: AP010958.1). For each of the organisms two sets of reads were generated, one of the Illumina platform and another of the 454 platform.

### Tool validation with simulated datasets of different coverage

A third validation step was performed, this time using simulated data with different sequencing coverage. The goal was to simulate different amounts of redundant reads by mimicking the PCR process. We selected as reference the genomes *Mycobacterium bovis* BCG str. Korea 1168P (dataset prefix name HS25MicoKorea1168P) *Mycobacterium tuberculosis* KZN 4207 (dataset prefix name HS25MicoKZN_4207) *and Escherichia coli* O103:H2 str. 12009 (dataset prefix name HS25EcoliO103_H2).

Each of the reference genomes was used in ART tool version 2.5.8 to generate simulated datasets with 100x, 200x and 300x coverage, respectively. Thus, nine simulated datasets were generated as shown in Table 6.

**Table 6:** Generation of simulated data with different coverage.

Organism	Coverage	Dataset Name
*Mycobacterium bovis* BCG str. Korea 1168P	300x	HS25MicoKorea1168P_300
*Mycobacterium bovis* BCG str. Korea 1168P	200x	HS25MicoKorea1168P_200
*Mycobacterium bovis* BCG str. Korea 1168P	100x	HS25MicoKorea1168P_100
*Mycobacterium tuberculosis* KZN 4207	300x	HS25MicoKZN_4207_300
*Mycobacterium tuberculosis* KZN 4207	200x	HS25MicoKZN_4207_200
*Mycobacterium tuberculosis* KZN 4207	100x	HS25MicoKZN_4207_100
*Escherichia coli* O103:H2 str. 12009	300x	HS25EcoliO103_H2_300
*Escherichia coli* O103:H2 str. 12009	200x	HS25EcoliO103_H2_200
*Escherichia coli* O103:H2 str. 12009	100x	HS25EcoliO103_H2_100

After this step we use an *ad-hoc* script (available in https://sourceforge.net/projects/ngsreadstreatment/files/AnalyzeDuplicatesInFastq.pl) designed to count the number of unique reads in a dataset, this is, reads that appear only once. The purpose of using this script was to determine if after processing the data, redundant reads were completely removed, thus ensuring that only unique reads would stay in each dataset. In this way, after each of the nine datasets were processed by each of the tools, the number of unique reads of each one was counted.

### Workstation

The Workstation used to carry out the analyzes has the following configuration: Intel Core i7-2620M CPU 2.70 GHz with four processing cores, 324 GB HD and 6GB memory.

## Supplementary information

List of all results about datasets processing
