# Supplementary material for: NGSReadsTreatment – A Cuckoo Filter-based Tool for Removing Duplicate Reads in NGS Data
Source: Sci Rep. 2019 Aug 12;9:11681. doi: 10.1038/s41598-019-48242-w (PMC6690869; doi:10.1038/s41598-019-48242-w)
Supplement: Supplementary file 1 — List of all results about datasets processing [file 41598_2019_48242_MOESM1_ESM.pdf]

NGSReadsTreatment – A Cuckoo Filter-based Tool for Removing Duplicate Reads in NGS Data

Antonio Sérgio Cruz Gaia[1+], Pablo Henrique Caracciolo Gomes de Sá[2+], Mônica Silva de Oliveira[1], Adonney Allan de Oliveira Veras[1*]

[1]Postgraduate Program in Applied Computing, Federal University of Pará (UFPA), Pará, Brazil

[2] Federal Rural University of Amazonia Campus Tomé-Açu (UFRA), Pará, Brazil

*allanveras@ufpa.br

+these authors contributed equally to this work

Supplementary Material

REAL DATA RESULTS

Organism	Library	File size in MB	Amount of reads			
Escherichia coli RR1	SRR2014554	8192	24.248.885			
**Tools**	**Processing time (in hours:minutes:seconds)**	**Total (s) number of CPU in Kernel Mode**	**Total (s) number of CPU in User Mode**	**Percentage of the CPU Usage**	**Total amount of memory Used in MB**	**Amount of reads After treatment**
**FastUniq 1.1**	Computer crashed after 40 min of processing					
**ParDRe 2.2.5**	Computer crashed after 17 min of processing					
**MarDre 1.3**	Error: Input files must be the same size in paired-end mode					
**CD-HIT-DUP 4.6.8**	After 01:03:53 Command exited with non-zero status 1.<br>Error: the pair end files contain different number of reads!					
**Clumpify (bbmap)**	After 00:07:25 Command exited with non-zero status 1.<br>There appear to be different numbers of reads in the paired input files.					
**NGSReadsTreatment**	00:20:13	526.63	746.84	104	549	24.179.406

**Library**	
Instrument	Illumina HiSeq 2000
Strategy	WGS
Source	GENOMIC
Selection	RANDOM
Layout	PAIRED

Organism	Library	File size in MB	Amount of reads			
Escherichia coli 042	ERR007646	2406	28.221.392			
**Tools**	**Processing time (in hours:minutes:seconds)**	**Total (s) number of CPU in Kernel Mode**	**Total (s) number of CPU in User Mode**	**Percentage of the CPU Usage**	**Total amount of memory Used in MB**	**Amount of reads After treatment**
**FastUniq 1.1**	00:01:24	3.66	30.12	40	3987	13.956.288
**ParDRe 2.2.5**	00:33:49	19.37	70.63	4	5387	13.956.364
**MarDre 1.3**	00:03:36	9.72	117.67	58	1653	13.956.288
**CD-HIT-DUP 4.6.8**	00:25:24	40.35	91.32	8	5393	13.956.288
**Clumpify (bbmap)**	00:03:25	11.22	103.08	55	870	13.927.100
**NGSReadsTreatment**	00:11:22	302.21	413.13	107	543	13.970.954

**Library**	
Name	no-PCR-sc-no-PCR-sc-E.coli-1-1
Instrument	Illumina Genome Analyzer
Strategy	OTHER
Source	GENOMIC
Selection	RANDOM
Layout	PAIRED

Organism	Library	File size in MB		Amount of reads		
Escherichia coli P12b	SRR2000272	1351		2.991.478		
**Tools**	**Processing time (in hours:minutes:seconds)**	**Total (s) number of CPU in Kernel Mode**	**Total (s) number of CPU in User Mode**	**Percentage of the CPU Usage**	**Total amount of memory Used in MB**	**Amount of reads After treatment**
**FastUniq 1.1**	00:00:25	1.53	8.29	38	1722	2.967.034
**ParDRe 2.2.5**	00:01:07	2.29	34.65	54	2278	2.967.266
**MarDre 1.3**	ERROR: Input files must be the same size in paired-end mode					
**CD-HIT-DUP 4.6.8**	00:01:12	3.37	24.72	38	3076	2.967.266
**Clumpify (bbmap)**	00:01:02	4.55	53.58	92	423	2.963.080
**NGSReadsTreatment**	00:02:24	58.57	91.27	103	537	2.934.404

**Library**	
Name	NexteraXT
Instrument	Illumina MiSeq
Strategy	WGS
Source	GENOMIC
Selection	RANDOM
Layout	PAIRED
Construction protocol	NexteraXT

Organism	Library	File size in MB		Amount of reads		
Escherichia coli KLY	SRR1424625	1682		6.866.668		
**Tools**	**Processing time (in hours:minutes:seconds)**	**Total (s) number of CPU in Kernel Mode**	**Total (s) number of CPU in User Mode**	**Percentage of the CPU Usage**	**Total amount of memory Used in MB**	**Amount of reads After treatment**
**FastUniq 1.1**	00:00:34	2.03	15.22	50	2571	6.866.668
**ParDRe 2.2.5**	00:01:14	3.22	41.39	59	3586	6.866.668
**MarDre 1.3**	00:02:34	8.29	80.50	57	1676	6.866.668
**CD-HIT-DUP 4.6.8**	00:01:21	4.12	33.49	56	4097	6.866.668
**Clumpify (bbmap)**	00:01:57	7.63	79.14	73	455	6.852.786
**NGSReadsTreatment**	00:05:26	138.91	206.63	105	539	6.802.266

**Library**	
Instrument	Illumina HiSeq 2000
Strategy	WGS
Source	GENOMIC
Selection	unspecified
Layout	PAIRED

Organism	Library	File size in MB		Amount of reads		
Escherichia coli O25b:H4-ST131	SRR933487	1070		3.214.312		
**Tools**	**Processing time (in hours:minutes:seconds)**	**Total (s) number of CPU in Kernel Mode**	**Total (s) number of CPU in User Mode**	**Percentage of the CPU Usage**	**Total amount of memory Used in MB**	**Amount of reads After treatment**
**FastUniq 1.1**	00:00:14	1.23	7.11	58	1449	3.191.014
**ParDRe 2.2.5**	00:00:24	1.77	21.87	95	1950	3.191.016
**MarDre 1.3**	00:00:55	4.86	44.13	87	1411	3.191.014
**CD-HIT-DUP 4.6.8**	00:00:22	1.97	14.99	76	2063	3.191.014
**Clumpify (bbmap)**	00:00:24	3.34	29.15	135	2215	3.178.716
**NGSReadsTreatment**	00:02:30	65.33	94.00	105	538	3.153.156

**Library**	
Instrument	Illumina Genome Analyzer IIx
Strategy	WGS
Source	GENOMIC
Selection	RANDOM
Layout	PAIRED
Layout	PAIRED

Organism	Library	File size in MB		Amount of reads		
Kineococcus rhizosphaerae DSM 19711	SRR6479489	2048		5.641.334		
**Tools**	**Processing time (in hours:minutes:seconds)**	**Total (s) number of CPU in Kernel Mode**	**Total (s) number of CPU in User Mode**	**Percentage of the CPU Usage**	**Total amount of memory Used in MB**	**Amount of reads After treatment**
**FastUniq 1.1**	00:00:50	2.39	14.47	33	2629	5.633.496
**ParDRe 2.2.5**	00:01:44	4.77	54.83	56	3629	5.633.504
**MarDre 1.3**	00:03:06	10.64	81.50	49	1652	5.633.496
**CD-HIT-DUP 4.6.8**	00:01:38	4.65	31.95	37	4313	5.633.496
**Clumpify (bbmap)**	00:02:36	9.84	74.34	53	3454	5.630.770
**NGSReadsTreatment**	00:04:37	110.41	173.28	102	538	5.572.908

**Library**	
Name	BSHNU
Instrument	Illumina HiSeq 2500
Strategy	WGS
Source	GENOMIC
Selection	RANDOM
Layout	PAIRED
Construction protocol	Regular (DNA)

Organism	Library	File size in MB		Amount of reads		
Kineococcus xinjiangensis DSM 22857	SRR6479482	2168		5.971.022		
**Tools**	**Processing time (in hours:minutes:seconds)**	**Total (s) number of CPU in Kernel Mode**	**Total (s) number of CPU in User Mode**	**Percentage of the CPU Usage**	**Total amount of memory Used in MB**	**Amount of reads After treatment**
**FastUniq 1.1**	00:01:43	4.45	19.19	22	2783	5.962.418
**ParDRe 2.2.5**	00:05:23	6.00	61.11	20	3850	5.962.424
**MarDre 1.3**	00:03:13	10.82	88.12	51	1647	5.962.418
**CD-HIT-DUP 4.6.8**	00:01.36	5.18	32.34	38	4501	5.962.418
**Clumpify (bbmap)**	00:02:52	10.45	79.49	52	3616	5.960.172
**NGSReadsTreatment**	00:05:01	122.05	191.75	104	538	5.900.978

**Library**	
Name	BSHNW
Instrument	Illumina HiSeq 2500
Strategy	WGS
Source	GENOMIC
Selection	RANDOM
Layout	PAIRED
Construction protocol	Regular (DNA)

Organism	Library	File size in MB	Amount of reads			
Mycobacterium tuberculosis F11	SRR974839	**1936**	14.110.696			
**Tools**	**Processing time (in hours:minutes:seconds)**	**Total (s) number of CPU in Kernel Mode**	**Total (s) number of CPU in User Mode**	**Percentage of the CPU Usage**	**Total amount of memory Used in MB**	**Amount of reads After treatment**
**FastUniq 1.1**	00:01:21	3.62	18.26	26	2725	7.233.122
**ParDRe 2.2.5**	00:09:39	4.87	534.85	93	3825	7.233.132
**MarDre 1.3**	00:10:47	11.39	462.76	73	1634	7.233.122
**CD-HIT-DUP 4.6.8**	00:01:44	5.43	36.38	40	4499	7.233.122
**Clumpify (bbmap)**	00:02:39	10.80	73.15	52	2625	7.186.020
**NGSReadsTreatment**	00:05:48	148.79	224.59	106	540	7.185.138

**Library**	
Name	Solexa-126846
Instrument	Illumina HiSeq 2000
Strategy	WGS
Source	GENOMIC
Selection	RANDOM
Layout	PAIRED

Organism	Library	File size in MB		Amount of reads		
Mycobacterium tuberculosis XDR KZN 4207	SRR1144800	1884		7.033.428		
**Tools**	**Processing time (in hours:minutes:seconds)**	**Total (s) number of CPU in Kernel Mode**	**Total (s) number of CPU in User Mode**	**Percentage of the CPU Usage**	**Total amount of memory Used in MB**	**Amount of reads After treatment**
**FastUniq 1.1**	00:01:20	2.98	16.53	24	2633	7.028.960
**ParDRe 2.2.5**	00:01:32	3.23	46.44	53	3730	7.028.960
**MarDre 1.3**	00:03:45	10.62	85.10	42	1653	7.028.960
**CD-HIT-DUP 4.6.8**	00:02:59	5.16	35.54	22	4351	7.028.960
**Clumpify (bbmap)**	00:03:26	10.19	68.26	38	3250	7.026.512
**NGSReadsTreatment**	00:05:30	140.15	209.60	105	540	6.967.590

**Library**	
Name	Pond-311521
Instrument	Illumina HiSeq 2000
Strategy	WGS
Source	GENOMIC
Selection	RANDOM
Layout	PAIRED

Organism	Library	File size in MB		Amount of reads		
Arcobacter halophilus strain CCUG 53805	SRR7587111	588		670.813		
**Tools**	**Processing time (in hours:minutes:seconds)**	**Total (s) number of CPU in Kernel Mode**	**Total (s) number of CPU in User Mode**	**Percentage of the CPU Usage**	**Total amount of memory Used in MB**	**Amount of reads After treatment**
**FastUniq 1.1**	Error in open left fastq file @SRR7587111.1 FZNUX9M02GJ4ET length=327 for read!					
**ParDRe 2.2.5**	00:00:23	0.91	15.77	72	888	668.356
**MarDre 1.3**	00:00:34	3.23	28.22	92	1118	668.356
**CD-HIT-DUP 4.6.8**	00:00:14	1.14	12.22	95	1561	668.356
**Clumpify (bbmap)**	00:00:13	1.93	24.38	192	769	667.959
**NGSReadsTreatment**	00:38:00	14.65	25.12	102	533	664.953

**Library**	
Name	AHALO454PE
Instrument	454 GS FLX+
Strategy	WGS
Source	GENOMIC
Selection	RANDOM
Layout	PAIRED

Organism	Library	File size in MB		Amount of reads		
Rhodopirellula baltica strain:BR-MGV	SRR7819959	1984		3.207.713		
**Tools**	**Processing time (in hours:minutes:seconds)**	**Total (s) number of CPU in Kernel Mode**	**Total (s) number of CPU in User Mode**	**Percentage of the CPU Usage**	**Total amount of memory Used in MB**	**Amount of reads After treatment**
**FastUniq 1.1**	Error in open left fastq file @SRR7819959.1 1 length=209 for read!					
**ParDRe 2.2.5**	00:01:30	3.39	62.44	72	3197	3.183.983
**MarDre 1.3**	00:02:42	10.02	87.13	59	1665	3.183.983
**CD-HIT-DUP 4.6.8**	cd-hit-dup: cdhit-dup.cxx:193: int HashingDepth(int, int): Assertion `len >= min' failed.					
**Clumpify (bbmap)**	00:02:17	6.31	90.72	70	659	3.181.912
**NGSReadsTreatment**	00:03:12	64.13	107.75	89	537	3.164.133

**Library**	
Name	Rhodopirellula baltica BR_MGV
Instrument	Ion Torrent PGM
Strategy	WGS
Source	GENOMIC
Selection	RANDOM
Layout	SINGLE

Organism	Library	File size in MB		Amount of reads		
Surveillance of Escherichia coli O157:H7 in Romania	ERR2375157	1201		2.106.268		
**Tools**	**Processing time (in hours:minutes:seconds)**	**Total (s) number of CPU in Kernel Mode**	**Total (s) number of CPU in User Mode**	**Percentage of the CPU Usage**	**Total amount of memory Used in MB**	**Amount of reads After treatment**
**FastUniq 1.1**	Error in open left fastq file @ERR2375157.1 1 length=294 for read!					
**ParDRe 2.2.5**	00:00:48	2.03	32.19	70	1989	2.104.786
**MarDre 1.3**	00:01:32	6.23	53.73	64	1394	2.104.786
**CD-HIT-DUP 4.6.8**	cd-hit-dup: cdhit-dup.cxx:193: int HashingDepth(int, int): Assertion `len >= min' failed.					
**Clumpify (bbmap)**	00:00:51	4.13	48.17	101	744	2.104.540
**NGSReadsTreatment**	00:02:05	42.26	70.90	89	537	2.073.554

**Library**	
Name	unspecified
Instrument	Ion Torrent PGM
Strategy	WGS
Source	GENOMIC
Selection	RANDOM
Layout	SINGLE

Organism	Library	File size in MB		Amount of reads		
Rathayibacter tritici strain:FH211, CT102, ATCC 11403 Raw sequence reads	SRR6799098	157		160.403		
**Tools**	**Processing time (in hours:minutes:seconds)**	**Total (s) number of CPU in Kernel Mode**	**Total (s) number of CPU in User Mode**	**Percentage of the CPU Usage**	**Total amount of memory Used in MB**	**Amount of reads After treatment**
**FastUniq 1.1**	Error in open left fastq file @SRR6799098.1 HTUX4QU01ANJ8O length=78 for read!					
**ParDRe 2.2.5**	00:00:06	0.25	4.16	70	247	157.022
**MarDre 1.3**	00:00:16	1.50	15.15	102	913	157.022
**CD-HIT-DUP 4.6.8**	00:00:03	0.27	3.33	99	481	157.022
**Clumpify (bbmap)**	00:00:02	0.30	5.31	208	373	156.842
**NGSReadsTreatment**	00:00:08	3.45	6.63	117	531	156.835

**Library**	
Name	FH211i
Instrument	454 GS Junior
Strategy	WGS
Source	GENOMIC
Selection	size fractionation
Layout	SINGLE

Organism	Library	File size in MB		Amount of reads		
Salmonella enterica subsp. enterica serovar Fresno strain:USMARC-69835	SRR7905974	2990		163.468		
**Tools**	**Processing time (in hours:minutes:seconds)**	**Total (s) number of CPU in Kernel Mode**	**Total (s) number of CPU in User Mode**	**Percentage of the CPU Usage**	**Total amount of memory Used in MB**	**Amount of reads After treatment**
**FastUniq 1.1**	Error in read from FASTQ file list!					
**ParDRe 2.2.5**	00:02:08	4.60	83.50	68	3796	163.464
**MarDre 1.3**	java.lang.Exception: java.lang.StringIndexOutOfBoundsException: String index out of range: 19					
**CD-HIT-DUP 4.6.8**	Computer crashed after 16 min of processing					
**Clumpify (bbmap)**	00:03:50	7.16	120.72	55	961	163.464
**NGSReadsTreatment**	00:02:08	8.20	33.51	32	526	163.249

**Library**	
Name	23060
Instrument	PacBio RS II
Strategy	WGS
Source	GENOMIC
Selection	RANDOM
Layout	SINGLE

Organism	Library	File size in MB		Amount of reads		
Staphylococcus aureus Cystic Fibrosis Isolates	SRR7739756	1336		86.389		
**Tools**	**Processing time (in hours:minutes:seconds)**	**Total (s) number of CPU in Kernel Mode**	**Total (s) number of CPU in User Mode**	**Percentage of the CPU Usage**	**Total amount of memory Used in MB**	**Amount of reads After treatment**
**FastUniq 1.1**	Error in read from FASTQ file list!					
**ParDRe 2.2.5**	00:00:57	2.34	35.76	66	1704	86.386
**MarDre 1.3**	INFO mapreduce.Job: Job job_local1848694102_0001 failed with state FAILED due to: NA					
**CD-HIT-DUP 4.6.8**	cd-hit-dup: cdhit-dup.cxx:193: int HashingDepth(int, int): Assertion `len >= min' failed.					
**Clumpify (bbmap)**	00:01:08	3.44	52.06	80	700	86.386
**NGSReadsTreatment**	00:00:17	3.35	13.64	95	532	86.324

**Library**	
Name	CFBR_EB_Sa105-MinION
Instrument	MinION
Strategy	WGS
Source	GENOMIC
Selection	RANDOM
Layout	SINGLE

Organism	Library	File size in MB		Amount of reads		
Pseudomonas aeruginosa microevolution during chronic infection of the CF lung	ERR2162181.fastq	246		1.146.696		
**Tools**	**Processing time (in hours:minutes:seconds)**	**Total (s) number of CPU in Kernel Mode**	**Total (s) number of CPU in User Mode**	**Percentage of the CPU Usage**	**Total amount of memory Used in MB**	**Amount of reads After treatment**
**FastUniq 1.1**	Error in open left fastq file @ERR2162181.1 1 length=75 for read					
**ParDRe 2.2.5**	00:00:09	0.43	6.78	73	625	1.146.694
**MarDre 1.3**	00:00:29	1.93	22.90	83	947	1.146.694
**CD-HIT-DUP 4.6.8**	00:00:04	0.58	4.34	99	779	1.146.694
**Clumpify (bbmap)**	Exception in thread "main" java.lang.AssertionError: ASCII encoding for quality (currently ASCII-33) appears to be wrong for input quality 15 for base T at lines 1 and 3, position 67.					
**NGSReadsTreatment**	00:00:53	22.87	35.42	108	535	1.142.899

**Library**	
Name	unspecified
Instrument	AB 5500xl Genetic Analyzer
Strategy	WGS
Source	GENOMIC
Selection	PCR
Layout	SINGLE

SIMULATED DATA RESULTS

Organism		Data Simulate	File size by Dataset		Total of Reads by Dataset		
Mycobacterium tuberculosis variant bovis BCG str. Korea 1168P			910MB		2,917,800		
**Number**	**Tools**	**Processing time (in hours:minutes:seconds)**	**Total (s) number of CPU in Kernel Mode**	**Total (s) number of CPU in User Mode**	**Percentage of the CPU Usage**	**Total amount of memory Used in MB**	**Amount of reads After treatment**
**1**	**FastUniq 1.1**	00:00:13	1.14	6.52	56	1272	2,915,606
**2**	**ParDRe 2.2.5**	The job has been aborted					
**3**	**MarDre 1.3**	00:00:57	5.35	45.13	88	1474	2,915,606
**4**	**CD-HIT-DUP 4.6.8**	00:00:43	2.28	14.08	37	2173	2,915,606
**5**	**Clumpify (bbmap)**	00:00:34	2.88	36.74	113	771	2,911,848
**6**	**NGSReadsTreatment**	00:02:19	58.79	90.63	107	537	2,884,114

Library

Name

Instrument Illumina HiSeq 2500

Strategy

Source

Selection

Layout PAIRED

Organism		Data Simulate	File size by Dataset		Total of Reads by Dataset		
Mycobacterium tuberculosis KZN 4207			914MB		2,929,900		
**Number**	**Tools**	**Processing time (in hours:minutes:seconds)**	**Total (s) number of CPU in Kernel Mode**	**Total (s) number of CPU in User Mode**	**Percentage of the CPU Usage**	**Total amount of memory Used in MB**	**Amount of reads After treatment**
**1**	**FastUniq 1.1**	00:00:12	1.15	6.57	60	1278	2,927,650
**2**	**ParDRe 2.2.5**	The job has been aborted					
**3**	**MarDre 1.3**	00:01:28	5.76	45.26	57	1533	2,927,650
**4**	**CD-HIT-DUP 4.6.8**	00:00:35	2.09	13.99	45	2153	2,927,650
**5**	**Clumpify (bbmap)**	00:00:28	2.74	39.03	148	538	2,923,922
**6**	**NGSReadsTreatment**	00:02:19	58.38	90.80	107	538	2,895,866

**Library**	
Name	
Instrument	Illumina HiSeq 2500
Strategy	
Source	
Selection	
Layout	PAIRED

Organism		Data Simulate	File size by Dataset		Total of Reads by Dataset		
Escherichia coli O103:H2 str. 12009			1132MB		3,632,800		
**Number**	**Tools**	**Processing time (in hours:minutes:seconds)**	**Total (s) number of CPU in Kernel Mode**	**Total (s) number of CPU in User Mode**	**Percentage of the CPU Usage**	**Total amount of memory Used in MB**	**Amount of reads After treatment**
**1**	**FastUniq 1.1**	00:00:17	1.42	8.08	53	1583	3,629,678
**2**	**ParDRe 2.2.5**	The job has been aborted					
**3**	**MarDre 1.3**	00:02:22	7.66	54.62	43	1632	3,629,678
**4**	**CD-HIT-DUP 4.6.8**	00:00:56	2.76	18.64	38	2660	3,629,678
**5**	**Clumpify (bbmap)**	00:00:46	3.82	46.82	108	558	3,624,308
**6**	**NGSReadsTreatment**	00:02:54	72.73	111.84	105	537	3,580,676

**Library**	
Name	
Instrument	Illumina HiSeq 2500
Strategy	
Source	
Selection	
Layout	PAIRED

Organism		Data Simulate	File size by Dataset		Total of Reads by Dataset		
Arcobacter halophilus strain CCUG 53805			588MB		1,875,000		
**Number**	**Tools**	**Processing time (in hours:minutes:seconds)**	**Total (s) number of CPU in Kernel Mode**	**Total (s) number of CPU in User Mode**	**Percentage of the CPU Usage**	**Total amount of memory Used in MB**	**Amount of reads After treatment**
**1**	**FastUniq 1.1**	00:00:05	0.74	4.29	89	832	1,873,432
**2**	**ParDRe 2.2.5**	The job has been aborted					
**3**	**MarDre 1.3**	00:00:51	4.15	32.80	72	1250	1,873,432
**4**	**CD-HIT-DUP 4.6.8**	00:00:12	1.12	8.19	76	1363	1,873,432
**5**	**Clumpify (bbmap)**	00:00:13	2.23	24.07	201	569	1,870,872
**6**	**NGSReadsTreatment**	00:01:27	35.45	59.45	108	536	1,860,626

**Library**	
Name	
Instrument	Illumina HiSeq 2500
Strategy	
Source	
Selection	
Layout	PAIRED

Organism		Data Simulate	File size by Dataset		Total of Reads by Dataset		
Mycobacterium tuberculosis variant bovis BCG str. Korea 1168P			104MB		251,688		
**Number**	**Tools**	**Processing time (in hours:minutes:seconds)**	**Total (s) number of CPU in Kernel Mode**	**Total (s) number of CPU in User Mode**	**Percentage of the CPU Usage**	**Total amount of memory Used in MB**	**Amount of reads After treatment**
**1**	**FastUniq 1.1**	00:00:00.63	0.16	0.46	99	143	251,682
**2**	**ParDRe 2.2.5**	The job has been aborted					
**3**	**MarDre 1.3**	Error: Input files must be the same size in paired-end mode					
**4**	**CD-HIT-DUP 4.6.8**	00:00:02	0.26	2.15	98	477	251,688
**5**	**Clumpify (bbmap)**	00:00:03	0.30	3.91	137	337	251,688
**6**	**NGSReadsTreatment**	00:00:17	8.13	11.29	109	534	251,436

**Library**	
Name	
Instrument	454
Strategy	
Source	
Selection	
Layout	PAIRED

Organism	Data Simulate	File size by Dataset		Total of Reads by Dataset		
Mycobacterium tuberculosis KZN 4207		104MB		252,954		

Number	Tools	Processing time (in hours:minutes:seconds)	Total (s) number of CPU in Kernel Mode	Total (s) number of CPU in User Mode	Percentage of the CPU Usage	Total amount of memory Used in MB	Amount of reads After treatment
**1**	**FastUniq 1.1**	00:00:00.62	0.14	0.48	100	143	252,954
**2**	**ParDRe 2.2.5**	The job has been aborted					
**3**	**MarDre 1.3**	Error: Input files must be the same size in paired-end mode					
**4**	**CD-HIT-DUP 4.6.8**	00:00:03	0.32	2.23	68	476	252,954
**5**	**Clumpify (bbmap)**	00:00:01	0.25	3.75	300	262	252,954
**6**	**NGSReadsTreatment**	00:00:12	5.23	8.84	115	534	252,748

**Library**	
Name	
Instrument	454
Strategy	
Source	
Selection	
Layout	PAIRED

Organism		Data Simulate	File size by Dataset		Total of Reads by Dataset		
Escherichia coli O103:H2 str. 12009			130MB		313,944		
**Number**	**Tools**	**Processing time (in hours:minutes:seconds)**	**Total (s) number of CPU in Kernel Mode**	**Total (s) number of CPU in User Mode**	**Percentage of the CPU Usage**	**Total amount of memory Used in MB**	**Amount of reads After treatment**
**1**	**FastUniq 1.1**	00:00:00.78	0.14	0.63	100	177	313,938
**2**	**ParDRe 2.2.5**	The job has been aborted					
**3**	**MarDre 1.3**	Error: Input files must be the same size in paired-end mode					
**4**	**CD-HIT-DUP 4.6.8**	00:00:04	0.46	2.66	65	598	313,944
**5**	**Clumpify (bbmap)**	00:00:01	0.37	4.18	265	400	313,944
**6**	**NGSReadsTreatment**	00:00:14	6.25	10.53	115	533	313,542

**Library**	
Name	
Instrument	454
Strategy	
Source	
Selection	
Layout	PAIRED

Organism		Data Simulate	File size by Dataset		Total of Reads by Dataset		
Arcobacter halophilus strain CCUG 53805			70MB		161,900		
**Number**	**Tools**	**Processing time (in hours:minutes:seconds)**	**Total (s) number of CPU in Kernel Mode**	**Total (s) number of CPU in User Mode**	**Percentage of the CPU Usage**	**Total amount of memory Used in MB**	**Amount of reads After treatment**
**1**	**FastUniq 1.1**	00:00:00.42	0.07	0.32	93	96	161,900
**2**	**ParDRe 2.2.5**	The job has been aborted					
**3**	**MarDre 1.3**	Error: Input files must be the same size in paired-end mode					
**4**	**CD-HIT-DUP 4.6.8**	0:00:02	0.17	1.47	65	321	161,900
**5**	**Clumpify (bbmap)**	00:00:01	0.21	3.03	166	264	161,900
**6**	**NGSReadsTreatment**	0:00:08	3.30	6.20	118	536	161,812

Library

Name

Instrument 454

Strategy

Source

Selection

Layout PAIRED

SIMULATED DATA WITH DIFFERENT COVERAGE VALUES

RESULTS

Organism		SRA Access number	File size by Dataset		Total of Reads by Dataset		
HS25EcoliO103_H2_100		HS25EcoliO103_H2_100	1132MB		3.632.800		
**Number**	**Tools**	**Processing time (in hours:minutes:seconds)**	**Total (s) number of CPU in Kernel Mode**	**Total (s) number of CPU in User Mode**	**Percentage of the CPU Usage**	**Total amount of memory Used in MB**	**Amount of reads After treatment**
**1**	**FastUniq 1.1**	00:00:20	1.47	8.51	47	1583	3.629.674
**2**	**ParDRe 2.2.5**	The job has been aborted					
**3**	**MarDre 1.3**	00:01:19	6.69	53.29	75	1619	3.629.674
**4**	**CD-HIT-DUP 4.6.8**	00:00:49	2.78	18.46	43	2695	3.629.674
**5**	**Clumpify (bbmap)**	00:01:06	4.76	54.45	88	462	3.624.414
**6**	**NGSReadsTreatment**	00:02:54	71.49	114.19	106	583	3.629.674

**Library**	
Name	
Instrument	HiSeq2500
Strategy	
Source	
Selection	
Layout	PAIRED

Organism	SRA Access number	File size by Dataset		Total of Reads by Dataset			
HS25MicoKorea1168P_100	HS25MicoKorea1168P_100	908MB		2.917.800			

Number	Tools	Processing time (in hours:minutes:seconds)	Total (s) number of CPU in Kernel Mode	Total (s) number of CPU in User Mode	Percentage of the CPU Usage	Total amount of memory Used in MB	Amount of reads After treatment
**1**	**FastUniq 1.1**	00:00:14	1.08	6.60	52	1273	2.915.548
**2**	**ParDRe 2.2.5**	The job has been aborted					
**3**	**MarDre 1.3**	00:01:30	5.60	44.85	55	1636	2.915.548
**4**	**CD-HIT-DUP 4.6.8**	00:00:39	2.16	14.17	41	2148	2.915.548
**5**	**Clumpify (bbmap)**	00:00:54	3.15	38.83	76	435	2.911.790
**6**	**NGSReadsTreatment**	00:02:30	58.81	93.24	101	571	2.915.548

**Library**	
Name	
Instrument	HiSeq2500
Strategy	
Source	
Selection	
Layout	PAIRED

Organism		SRA Access number	File size by Dataset		Total of Reads by Dataset		
HS25MicoKZN_4207_100		HS25MicoKZN_4207_100	914MB		2.929.900		
**Number**	**Tools**	**Processing time (in hours:minutes:seconds)**	**Total (s) number of CPU in Kernel Mode**	**Total (s) number of CPU in User Mode**	**Percentage of the CPU Usage**	**Total amount of memory Used in MB**	**Amount of reads After treatment**
**1**	**FastUniq 1.1**	00:00:24	1.42	6.88	33	1278	2.927.610
**2**	**ParDRe 2.2.5**	The job has been aborted					
**3**	**MarDre 1.3**	00:01:20	5.68	45.61	63	1465	2.927.610
**4**	**CD-HIT-DUP 4.6.8**	00:00:35	2.07	14.04	45	2155	2.927.610
**5**	**Clumpify (bbmap)**	00:00:30	2.65	39.96	139	451	2.923.848
**6**	**NGSReadsTreatment**	00:02:17	56.57	90.82	106	573	2.927.610

**Library**	
Name	
Instrument	HiSeq2500
Strategy	
Source	
Selection	
Layout	PAIRED

Organism		SRA Access number	File size by Dataset		Total of Reads by Dataset		
HS25EcoliO103_H2_200		HS25EcoliO103_H2_200	2266MB		7.265.600		
**Number**	**Tools**	**Processing time (in hours:minutes:seconds)**	**Total (s) number of CPU in Kernel Mode**	**Total (s) number of CPU in User Mode**	**Percentage of the CPU Usage**	**Total amount of memory Used in MB**	**Amount of reads After treatment**
**1**	**FastUniq 1.1**	00:01:33	3.79	18.55	23	3163	7.253.374
**2**	**ParDRe 2.2.5**	The job has been aborted					
**3**	**MarDre 1.3**	00:06:58	16.93	98.24	27	1660	7.253.374
**4**	**CD-HIT-DUP 4.6.8**	00:04:59	6.35	39.81	15	5260	7.253.374
**5**	**Clumpify (bbmap)**	00:03:28	9.95	118.71	61	405	7.232.320
**6**	**NGSReadsTreatment**	00:06:11	147.42	224.89	100	625	7.253.374

**Library**	
Name	
Instrument	HiSeq25
Strategy	
Source	
Selection	
Layout	PAIRED

Organism		SRA Access number	File size by Dataset		Total of Reads by Dataset		
HS25MicoKorea1168P_200		HS25MicoKorea1168P_200	1814MB		5.835.600		
**Number**	**Tools**	**Processing time (in hours:minutes:seconds)**	**Total (s) number of CPU in Kernel Mode**	**Total (s) number of CPU in User Mode**	**Percentage of the CPU Usage**	**Total amount of memory Used in MB**	**Amount of reads After treatment**
**1**	**FastUniq 1.1**	00:01:11	2.72	14.89	24	2541	5.826.662
**2**	**ParDRe 2.2.5**	The job has been aborted					
**3**	**MarDre 1.3**	00:04:09	11.59	82.91	37	1674	5.826.662
**4**	**CD-HIT-DUP 4.6.8**	00:01:25	4.36	29.67	40	4333	5.826.662
**5**	**Clumpify (bbmap)**	Out Of Memory Error					
**6**	**NGSReadsTreatment**	00:05:05	121.96	188.88	101	609	5.826.662

**Library**	
Name	
Instrument	HiSeq2500
Strategy	
Source	
Selection	
Layout	PAIRED

Organism	SRA Access number	File size by Dataset	Total of Reads by Dataset
HS25MicoKZN_4207_200	HS25MicoKZN_4207_200	1828MB	5.859.800

Number	Tools	Processing time (in hours:minutes:seconds)	Total (s) number of CPU in Kernel Mode	Total (s) number of CPU in User Mode	Percentage of the CPU Usage	Total amount of memory Used in MB	Amount of reads After treatment
**1**	**FastUniq 1.1**	00:01:11	3.10	15.38	25	2552	5.850.850
**2**	**ParDRe 2.2.5**	The job has been aborted					
**3**	**MarDre 1.3**	00:04:44	11.32	80.51	32	1607	5.850.850
**4**	**CD-HIT-DUP 4.6.8**	00:01:27	4.80	30.16	40	4353	5.850.850
**5**	**Clumpify (bbmap)**	Out Of Memory Error					
**6**	**NGSReadsTreatment**	00:05:02	119.56	188.09	101	610	5.850.850

Library

Name
Instrument: HiSeq 2500
Strategy
Source
Selection
Layout PAIRED

Organism		SRA Access number	File size by Dataset		Total of Reads by Dataset		
HS25EcoliO103_H2_300		HS25EcoliO103_H2_300	3400MB		10.898.400		
**Number**	**Tools**	**Processing time (in hours:minutes:seconds)**	**Total (s) number of CPU in Kernel Mode**	**Total (s) number of CPU in User Mode**	**Percentage of the CPU Usage**	**Total amount of memory Used in MB**	**Amount of reads After treatment**
**1**	**FastUniq 1.1**	00:02:34	6.21	31.04	24	4743	10.871.060
**2**	**ParDRe 2.2.5**	The job has been aborted					
**3**	**MarDre 1.3**	00:09:22	26.03	144.80	30	1658	10.871.060
**4**	**CD-HIT-DUP 4.6.8**	Computer crashed after 58 min of processing					
**5**	**Clumpify (bbmap)**	00:06:10	15.65	180.36	52	469	10.824.528
**6**	**NGSReadsTreatment**	00:09:38	232.77	357.18	101	671	10.871.060

**Library**	
Name	
Instrument	HiSeq2500
Strategy	
Source	
Selection	
Layout	PAIRED

Organism		SRA Access number	File size by Dataset		Total of Reads by Dataset		
HS25MicoKorea1168P_300		HS25MicoKorea1168P_300	2722MB		8.753.400		
**Number**	**Tools**	**Processing time (in hours:minutes:seconds)**	**Total (s) number of CPU in Kernel Mode**	**Total (s) number of CPU in User Mode**	**Percentage of the CPU Usage**	**Total amount of memory Used in MB**	**Amount of reads After treatment**
**1**	**FastUniq 1.1**	00:01:19	3.70	22.74	33	3810	8.733.052
**2**	**ParDRe 2.2.5**	The job has been aborted					
**3**	**MarDre 1.3**	00:06:26	17.77	114.96	34	1636	8.733.052
**4**	**CD-HIT-DUP 4.6.8**	Computer crashed after 15 min of processing					
**5**	**Clumpify (bbmap)**	00:03:39	10.95	134.95	66	416	8.699.550
**6**	**NGSReadsTreatment**	00:07:31	178.79	271.85	99	647	8.733.052

**Library**	
Name	
Instrument	HiSeq2500
Strategy	
Source	
Selection	
Layout	PAIRED

Organism	SRA Access number	File size by Dataset	Total of Reads by Dataset
HS25MicoKZN_4207_300	HS25MicoKZN_4207_300	2742MB	8.789.700

Number	Tools	Processing time (in hours:minutes:seconds)	Total (s) number of CPU in Kernel Mode	Total (s) number of CPU in User Mode	Percentage of the CPU Usage	Total amount of memory Used in MB	Amount of reads After treatment
**1**	**FastUniq 1.1**	00:03:24	6.53	30.32	18	3826	8.769.474
**2**	**ParDRe 2.2.5**	The job has been aborted					
**3**	**MarDre 1.3**	00:06:08	17.83	112.64	35	1658	8.769.474
**4**	**CD-HIT-DUP 4.6.8**	Computer crashed after 17 min of processing					
**5**	**Clumpify (bbmap)**	00:03:55	12.74	137.06	63	533	8.735.712
**6**	**NGSReadsTreatment**	00:07:45	185.19	278.90	99	647	8.769.474

Library

Name	
Instrument	HiSeq2500
Strategy	
Source	
Selection	
Layout	PAIRED

		FastUniq 1.1		ParDRe 2.2.5		MarDre 1.3		CD-HIT-DUP 4.6.8		Clumpify (bbmap)		NGSReadsTreatment	
Dataset Name	Amount of reads by dataset	Memory Usage	Amount of unique reads	Memory Usage	Amount of unique reads	Memory Usage	Amount of unique reads	Memory Usage	Amount of unique reads	Memory Usage	Amount of unique reads	Memory Usage	Amount of unique reads
HS25MicoKorea1168P_300	8753400	3810	8733052	NP	NP	1636	8733052	NP	NP	416	8.699.550	647	8733052
HS25MicoKorea1168P_200	5835600	2541	5826662	NP	NP	1674	5826662	4333	5826662	NP	NP	609	5826662
HS25MicoKorea1168P_100	2917800	1273	2915548	NP	NP	1636	2915548	2148	2915548	435	2911790	571	2915548
HS25MicoKZN_4207_300	8789700	3826	8769474	NP	NP	1658	8769474	NP	NP	533	8735712	647	8769474
HS25MicoKZN_4207_200	5859800	2552	5850850	NP	NP	1607	5850850	4353	5850850	NP	NP	610	5850850
HS25MicoKZN_4207_100	2929900	1278	2927610	NP	NP	1465	2927610	2155	2927610	451	2923848	573	2927610
HS25EcoliO103_H2_300	10898400	4743	10871060	NP	NP	1658	10871060	NP	NP	469	10824528	671	10871060
HS25EcoliO103_H2_200	7265600	3163	7253374	NP	NP	1660	7253374	5260	7253374	405	7232320	625	7253374
HS25EcoliO103_H2_100	3632800	1583	3629674	NP	NP	1619	3629674	2695	3629674	462	3624414	583	3629674
